# gma‐miR828a Negatively Regulates Resistance to Tea Leaf Spot Caused by *Lasiodiplodia theobromae* Through Targeting the CsMYB28– *CsRPP13* Module

**DOI:** 10.1111/mpp.70069

**Published:** 2025-03-03

**Authors:** Yuxuan Wen, Tianxinyi Pan, Yuancan Shi, Jinhui Xu, Delu Wang, Jing‐Jiang Zhou, Baoan Song, Zhuo Chen

**Affiliations:** ^1^ State Key Laboratory of Green Pesticides Guizhou University Guiyang China; ^2^ College of Forestry Guizhou University Guiyang China

**Keywords:** *CsMYB28*, *CsRPP13*, gene function, gma‐miR828a, *Lasiodiplodia theobromae*, resistance mechanism, tea leaf spot

## Abstract

Leaf spot caused by the fungus *Lasiodiplodia theobromae* severely affects the quality and production of tea ( *Camellia sinensis* ) in plantations across southwestern China. Currently, no effective control measures are available, and the damage to tea leaves is also exacerbated by a lack of understanding regarding the epidemiology of the disease. Previous studies have suggested that gma‐miR828a is differentially expressed during *L. theobromae* infection and may target and cleave the mRNA of *CsMYB28*. In this study, we characterised *CsMYB28* as encoding a transcription factor (TF) that localises to the nucleus, cell membrane, and cytoplasm. This gene was found to be differentially and spatiotemporally expressed in leaf tissues following *L. theobromae* infection of leaves of the tea plant. Altered *CsMYB28* expression, achieved by transient overexpression or stable genetic transformation of *Nicotiana benthamiana*, or transient silencing using antisense oligonucleotides (AsODN) in the tea plant, indicated that *CsMYB28* contributes to resistance against *L. theobromae*. Using DNA affinity purification sequencing, yeast one‐hybrid, and dual‐luciferase assays, we also identified that *CsMYB28* bound to the AATTAATT motif of *CsRPP13*, thereby activating the expression of *CsRPP13*. Additionally, degradome sequencing, β‐glucuronidase (GUS) assays, and RNA ligase‐mediated rapid amplification of cDNA ends revealed that miR828a cleaved *CsMYB28* mRNA, negatively regulating its expression. The results from transient overexpression and stable transformation studies, combined with AsODN‐mediated silencing in the tea plant, suggested that miR828a plays a negative regulatory role in modulating the response of the tea plant to *L. theobromae* infection. This study demonstrates that the miR828a–*CsMYB28*–*CsRPP13* mediates the response of the tea plant to *L. theobromae* infection.

## Graphical abstract

The tea plant transcription factor CsMYB28, which is targeted and regulated by gma‐miR828a, activates the expression of *CsRPP13*, thereby controlling tea leaf spot caused by *Lasiodiplodia theobromae*.

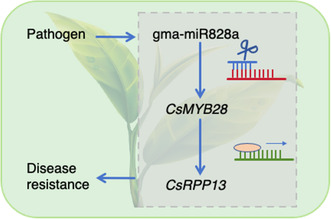

## 1 Introduction

Tea is an important economic crop, widely cultivated in over 30 countries (Lu et al. 2024; Zhao et al. 2011). Tea bushes are perennial plants that thrive in warm, rainy, and humid areas, which create conditions conducive to the development of diseases (Li et al. 2022; Lv et al. 2024). Studies have shown that there are numerous different tea diseases that can affect various parts of the tea bush, including the roots, stems, leaves, and flowers, thereby impacting the growth of the trees, as well as the yield and quality of the tea leaves (Pandey et al. 2021; Wang et al. 2019, 2020; Yin, Jiang, et al. 2021). It has been reported that there are over 500 species of tea pathogens worldwide, with more than 140 species occurring in China (Pandey et al. 2021; Yin, Yang, et al. 2021). Tea leaf spot, caused by *Lasiodiplodia theobromae*, is a recently discovered significant foliar disease. It is particularly prevalent in hot and humid environmental conditions. Due to the lack of understanding of the epidemiology of this disease and the lack of safe and effective control measures, tea leaf spot caused by *L. theobromae* continues to have a notable impact on both the yield and quality of tea leaves, which has become a major challenge to control this pathogen in tea‐producing regions of southwestern China in recent years (Li et al. 2021).

Studies have shown that the interaction between mRNA and non‐coding RNAs (ncRNAs) can confer resistance traits to tea plants (Jeyaraj et al. 2019; Wang, Liu, et al. 2021). ncRNAs, including long non‐coding RNA (lncRNA), microRNA (miRNA), and circular RNA (circRNA), are often considered a distinct special class of RNA that lacks protein‐coding capabilities (David 2004; Palos et al. 2023; Yu et al. 2019). They play an indispensable role in processes such as growth and development, cell differentiation, and response to both abiotic and biotic stresses (Feng et al. 2014; Samarfard et al. 2022; Wang et al. 2017).

Within plants, miRNAs can target and cleave mRNA. Once mRNA is degraded in this way, it loses its protein‐coding function. With advances in high‐throughput sequencing technology, an increasing number of miRNAs responsive to biological stresses have been discovered and studied. Conserved miRNA sequences are also involved in a range of important processes in plant growth and development, including the regulation of leaf morphogenesis, organ differentiation and development, and biosynthesis and metabolism, as well as disease response (Aukerman and Sakai 2003; Guo et al. 2005; Liu et al. 2012; Schwab et al. 2005; Sun et al. 2017; Xu et al. 2020; Zhao et al. 2018).

Some studies have reported that miRNAs in rice, maize, and tomato are involved in defence against pathogens (Wang et al. 2019). However, reports from tea research remain relatively scarce, although research indicates that miRNAs in tea plants may play a role in disease resistance. For example, csn‐miR477 can target and suppress the expression of phenylalanine ammonia‐lyase, increasing sensitivity to *Candida albicans* and promoting fungal infection (Wang et al. 2020). Additionally, miR319a inhibits the expression of the tea plant transcription factor (TF) gene *TCP10*, thereby enhancing infection by *Pestalotiopsis* sp. (Liu et al. 2022).

MYB TFs are widely distributed across higher plants and represent one of the largest families of TFs. They play crucial roles in regulating secondary metabolism, plant growth and development, hormone signal transduction, and stress responses. MYB TFs primarily regulate the expression of key downstream genes involved in defence responses as well as plant growth and development (Ma et al. 2023).

Our research group has previously generated full transcriptome‐sequencing data from tea plant leaf tissues infected by *L. theobromae* (Guo et al. 2022). Through differential expression and bioinformatics analyses, we identified gma‐miR828a and its potential target gene *CsMYB28*; the current study focuses on this relationship and investigates the regulatory mechanisms and functional role in disease resistance. The findings will support research on miRNA responses to biotic stresses and on TFs regulating tea plant growth and stress resistance/tolerance, providing valuable data for use in the breeding of disease‐resistant tea cultivars.

## 2 Results

### 2.1 CsMYB28 Functions as a TF in Tea Plants

**FIGURE 1:**
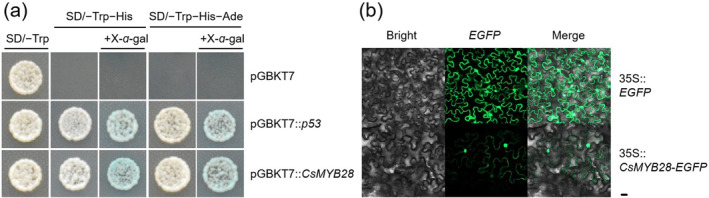
Assessment of the transcriptional activation activity of CsMYB28 and its subcellular localisation analysis. (a) Self‐activation assay of CsMYB28. After *CsMYB28* was ligated to the pGBKT7 vector and transformed into the AH109 yeast, the yeast was cultured for 3 days on synthetic defined (SD) selective media SD/−Trp, SD/−Trp/−His, and SD/−Trp/−His/−Ade plates, with pGBKT7 as the negative control and pGBKT7::*p53* as the positive control. Colourimetric reaction of the *β*‐galactosidase reporter gene system occurred with X‐*α*‐gal. (b) Subcellular localisation of CsMYB28 in *Nicotiana benthamiana* leaves. The fusion protein (35S::*CsMYB28‐GFP*) and 35S::*GFP* positive control were transiently expressed in *N. benthamiana* leaves for 2 days. Images were captured in a dark field to observe green fluorescence, while the cell outlines and the merged images were obtained in a bright field using a confocal microscope (LSM 900; Zeiss). Scale bar = 20 μm.

A phylogenetic analysis of CsMYB28 revealed that it clustered with *Arabidopsis* PRODUCTION OF FLAVONOL GLYCOSIDES 1, suggesting its potential involvement in flavonoid biosynthesis and host disease resistance (Figure S1). To verify its transcriptional activation ability, the pGBKT7‐*CsMYB28* vector was constructed and then transformed into competent yeast cells. The results demonstrated that CsMYB28 acted as a transcriptional activator (Figure 1a). The subcellular localisation studies showed that CsMYB28 was localised in the nucleus, cell membrane, and cytoplasm (Figure 1b). These findings suggested that CsMYB28 is a TF in tea plants.

### 2.2 *CsMYB28* Responds to the Infection of Tea Leaves by *L. theobromae*

**FIGURE 2:**
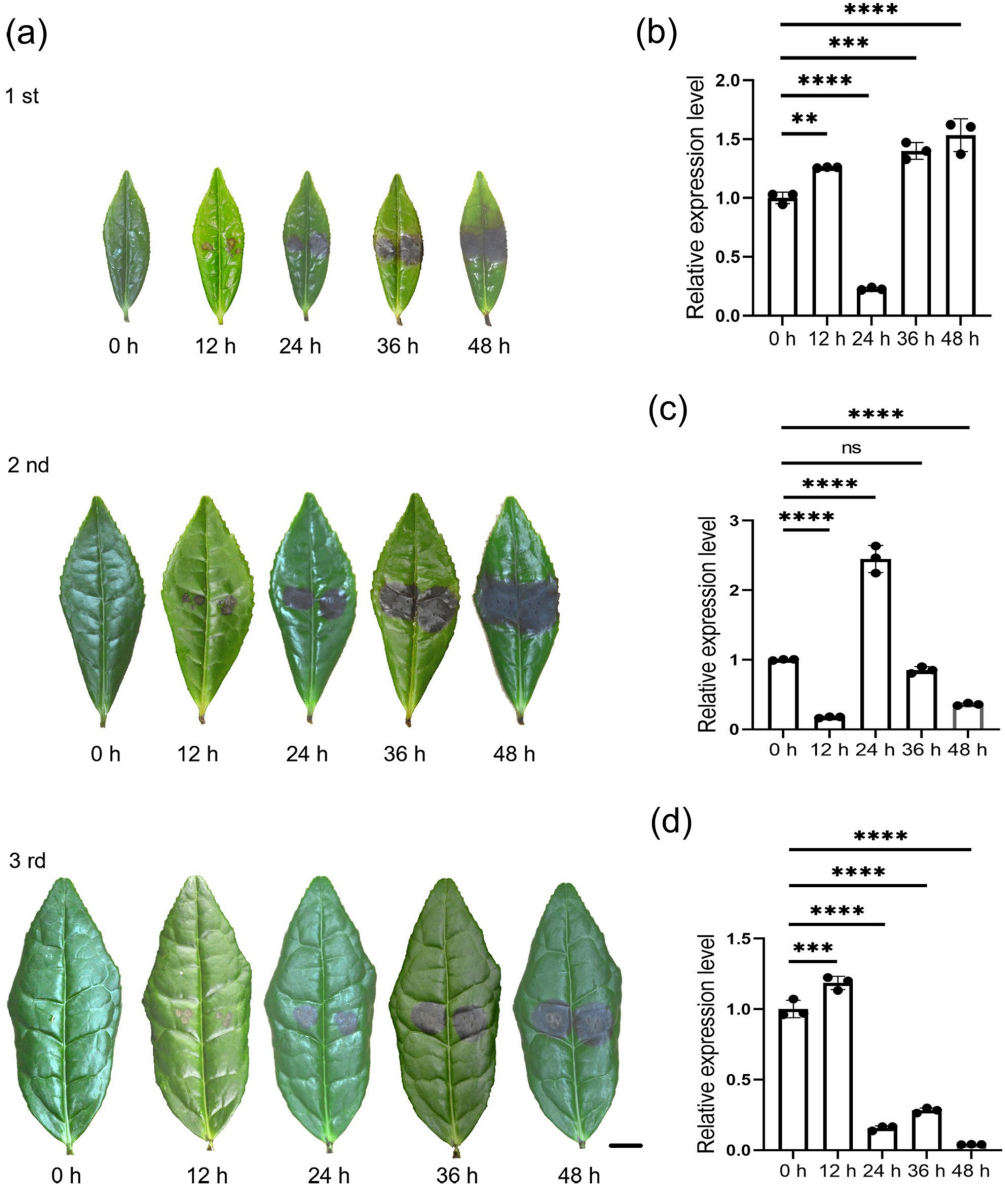
Spatiotemporal expression of *CsMYB28*. (a) The detached tea leaves were inoculated with *Lasiodiplodia theobromae* hyphal plugs for 12, 24, 36, and 48 h. Leaf samples were extracted at each time point and the expression of *CsMYB28* was determined using reverse transcription‐quantitative PCR. Scale bar = 1 cm. (b) The first leaf on tea plant shoots was treated as described in (a). (c) The second leaf on tea plant shoots was treated as described in (a). (d) The third leaf on tea plant shoots was treated as described in (a). Data are expressed as mean ± SD (*n* = 3). Statistical significance was determined using Student's *t* test (ns *p* > 0.05, **p < 0.01, ***p < 0.001, ****p < 0.0001).

In order to study the expression level of *CsMYB28* in tea leaves of different ages and at different times after *L. theobromae* inoculation, detached tea leaves, ranging in age from the first to the third leaves on a shoot, were subjected to testing with hyphal plugs from cultures of *L. theobromae* from 0 to 48 h after inoculation (Figure 2a). Reverse transcription‐quantitative PCR (RT‐qPCR) analysis revealed that the expression trend of *CsMYB28* varied among different leaves and inoculation times (Figure 2b–d). Specifically, *CsMYB28* exhibited an overall trend of up‐regulated expression in response to time after inoculation on the first leaf, except for 24 h after inoculation (Figure 2b). With the exception of 24 h after inoculation, *CsMYB28* expression also exhibited a down‐regulated expression trend with time after inoculation on the second leaf (Figure 2c), whereas for the third tea leaf, *CsMYB28* expression exhibited a down‐regulated trend with time after inoculation, except for the sample at 12 h after inoculation (Figure 2d).

### 2.3 *CsMYB28* Positively Regulates Disease Resistance in Tea Plants

**FIGURE 3:**
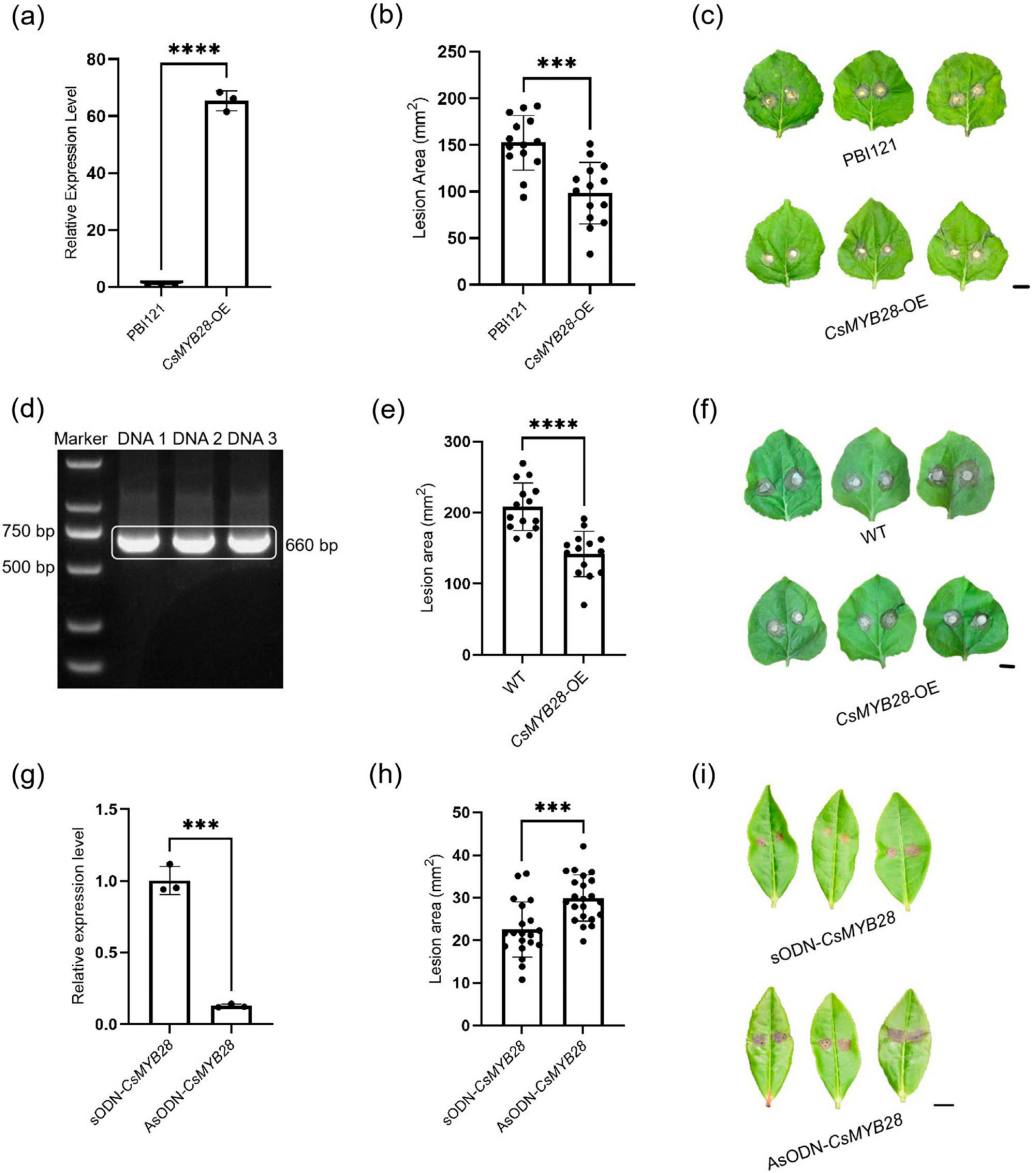
Effects of *CsMYB28* on disease resistance. (a) Relative expression levels of *CsMYB28* in pBI121 and 35S:*CsMYB28* overexpressing (*CsMYB28*‐OE) *Nicotiana benthamiana* leaves 2 days after transfection, as determined by reverse transcription‐quantitative PCR. (b) Lesion areas on *N. benthamiana* leaves 2 days post‐transfection with either pBI121 empty vector or *CsMYB28*‐OE, followed by 40 h post‐inoculation with hyphal plugs of *Botrytis cinerea*. (c) Disease symptoms in leaves transfected with pBI121 or *CsMYB28*‐OE were observed 40 h post‐inoculation with hyphal plugs of *B. cinerea* . (d) *CsMYB28*‐OE transgenic *N. benthamiana* leaves were verified by PCR using specific primers. (e) Lesion areas on wild‐type *N. benthamiana* leaves and *CsMYB28*‐OE transgenic *N. benthamian*a leaves were observed 40 h post‐inoculation with *B. cinerea* hyphae. (f) Virulence of *B. cinerea* hyphae on *CsMYB28*‐OE transgenic *N. benthamiana* leaves, with wild‐type *N. benthamiana* as a control, was assessed based on lesion size 40 h post‐inoculation. (g) The relative expression level of *CsMYB28* in antisense oligonucleotide (AsODN)‐treated and sense oligonucleotide (sODN)‐treated leaves, using RT‐qPCR. (h) The lesion areas on AsODN‐*CsMYB28*‐treated and sODN‐*CsMYB28*‐treated leaves were observed 1 day post‐inoculation with *Lasiodiplodia theobromae* hyphae. (i) The disease symptoms of AsODN‐*CsMYB28*‐treated and sODN‐*CsMYB28*‐treated tea leaves 1 day after inoculation with *L*. *theobromae* hyphae. All scale bars = 1 cm. Data are expressed as mean ± SD (*n*  ≥ 3). Statistical significance was determined using Student's *t* test (***p < 0.001, ****p < 0.0001).

To investigate the role of *CsMYB28* in tea leaves in resistance against *L. theobromae*, a 35S:*CsMYB28* vector was constructed to achieve transient overexpression of the gene in *Nicotiana benthamiana* leaves; RT‐qPCR confirmed that *CsMYB28* expression was up‐regulated in the overexpressing line relative to the pBI121 control (Figure 3a). A virulence test with *Botrytis cinerea* hyphal plugs showed that the lesion area was significantly reduced in leaves transiently overexpressing *CsMYB28* compared with the control leaves (Figure 3b,c).

In addition, the *CsMYB28‐*overexpressing transgenic *N. benthamiana* plants, generated with the 35S:*CsMYB28* vector, exhibited increased resistance to *B. cinerea* (Figure 3d). The virulence test demonstrated that the transgenic plants had significantly smaller lesions compared with the wild‐type control plants (Figure 3e,f).

To further investigate the impact of *CsMYB28* on disease resistance, antisense oligonucleotides (AsODN) were shown to successfully suppress the expression of the target gene, *CsMYB28*, in tea leaves, with RT‐qPCR results confirming that AsODN down‐regulated the expression of *CsMYB28* by 87.0% (Figure 3g). Inoculation of detached tea leaves with *L. theobromae* hyphal plugs showed that leaves treated with *CsMYB28*‐AsODN produced significantly larger lesion areas than those treated with the corresponding sense oligonucleotides (sODN, i.e., the *CsMYB28*‐sODN treatment) (Figure 3h,i). Both overexpression and silencing experiments suggested that *CsMYB28* plays a significant role in disease resistance.

### 2.4 DAP‐Seq on CsMYB28

**FIGURE 4:**
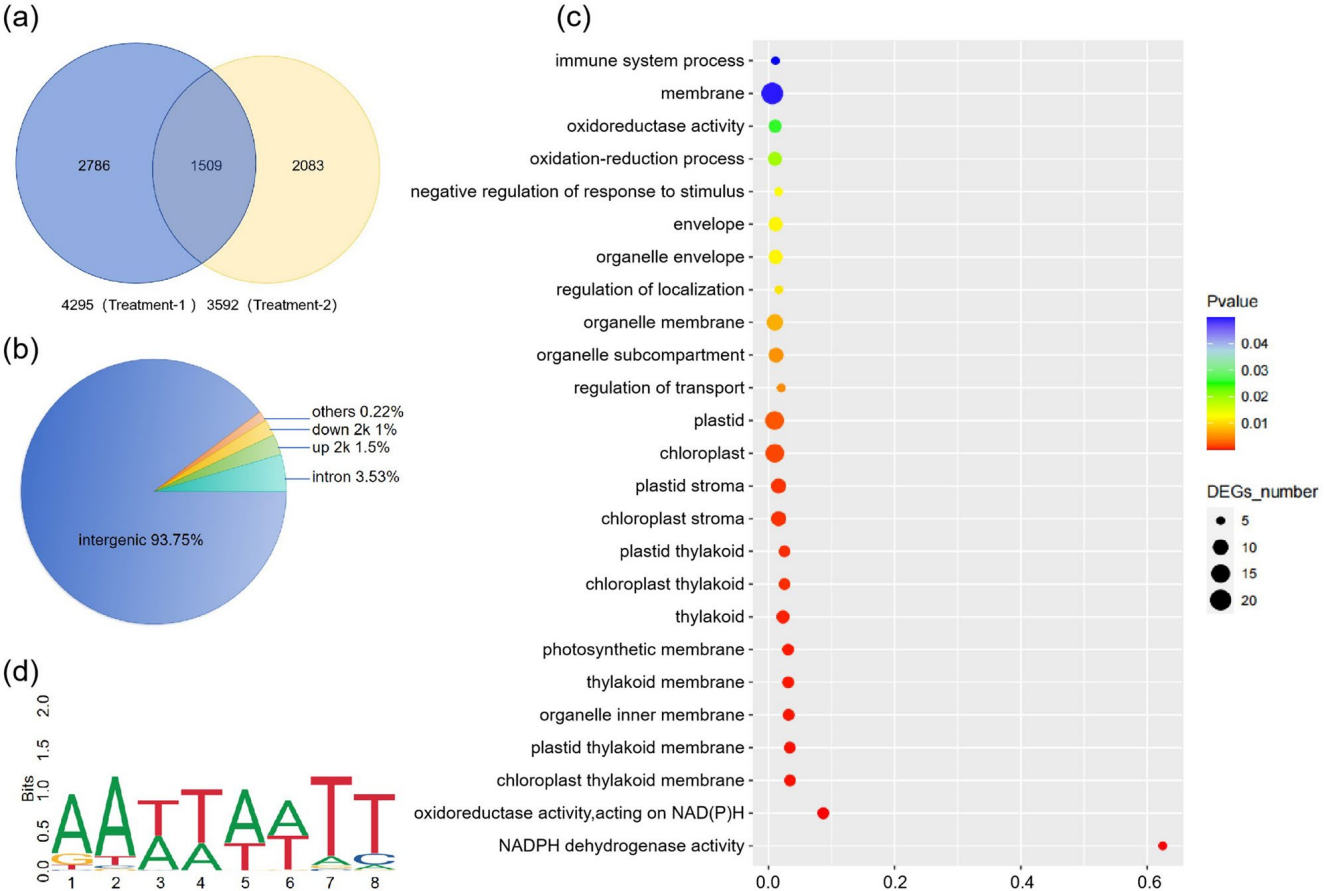
DNA affinity purification sequencing (DAP‐seq) data analysis. (a) Venn diagram showing the number of binding sites for CsMYB28 between the two treatments. (b) Statistics on the distribution of binding sites for CsMYB28. (c) Gene Ontology (GO) enrichment scatter plot of the target genes of CsMYB28. (d) Predicted binding motifs of CsMYB28 to the promoter *CsRPP13*.

The DNA affinity purification‐sequencing (DAP‐seq) assay and a TF‐binding site discovery assay indicated that CsMYB28 could potentially bind to 6378 putative binding regions, that is, peaks (Figure 4a). The CsMYB28‐binding regions located in the 2‐kb upstream region occupied 1.5% of all binding regions (Figure 4b). The analysis of peak lengths revealed that all peak lengths were within 2500 bp, with the majority falling within the 200–300 bp range, accounting for 44.2% (Figure S2). Subsequently, the frequency distribution of CsMYB28‐binding regions in the 10‐kb region upstream and downstream of the transcription start site (TSS) was determined. CsMYB28 was shown to prefer binding to DNA sequences in the vicinity of the TSS, which were highly enriched within the 300–500 bp of the promoter region (Figure S3). Therefore, CsMYB28 tended to bind to DNA elements in the promoter region to regulate gene expression. The enrichment analysis of the differentially expressed genes (DEGs) binding CsMYB28 indicated that CsMYB28 could regulate a range of cellular life activities and participate in a variety of biological functions, such as oxidoreductase activity, acting on NAD(P)H, chloroplast thylakoid membrane, plastid thylakoid membrane, thylakoid, chloroplast (Figure 4c). Interestingly, the DAP‐seq data showed that CsMYB28 directly targeted the disease‐resistance gene *CsRPP13* through the binding motif AATTAATT, suggesting that it might be involved in the plant defence response by regulating this disease‐resistance gene (Figure 4d).

### 2.5 CsMYB28 Regulates Expression of the Disease‐Resistance Gene *CsRPP13* to Induce Plant Defence

**FIGURE 5:**
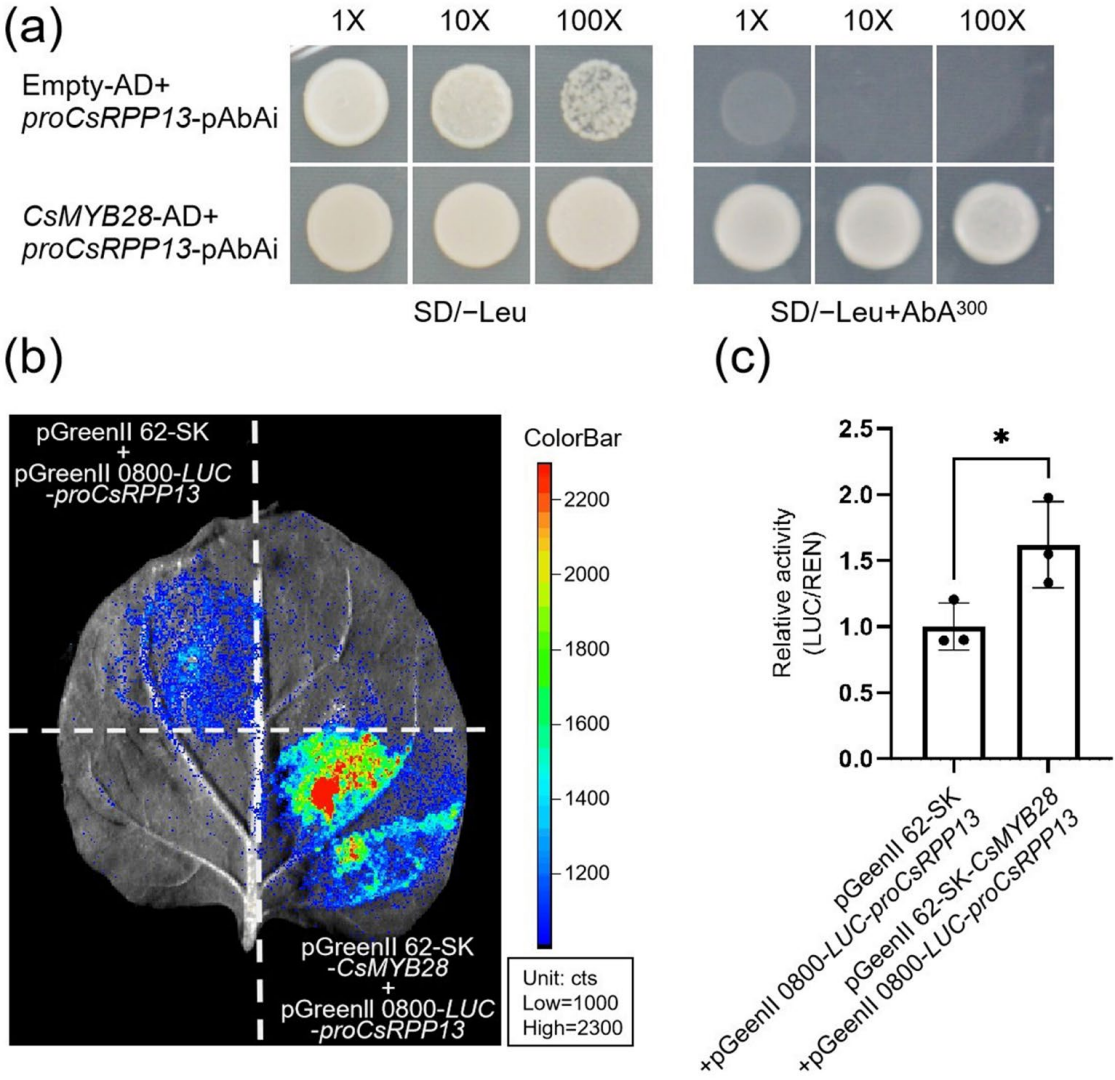
Yeast one‐hybrid (Y1H) and dual‐luciferase assays. (a) Y1H assay. Yeast cells containing pGADT7 empty vector + pro*CsRPP13‐*pAbAi or *CsMYB28*‐pGADT7 + pro*CsRPP13‐*pAbAi were transformed and grown in synthetic defined SD/−Leu medium. Growth was observed on the selective medium containing 0, 50, 150, 300, and 500 ng/mL of aureobasidin A (AbA). (b) pGreenII 62‐SK‐*CsMYB28* + pGreen II 0800‐*LUC*‐*proCsRPP13*, containing effector and reporter genes, respectively, were infiltrated into *Nicotiana benthamiana* leaves, and the different regions in the same *N. benthamiana* leaf blade were injected with pGreenII 62‐SK empty vector + pGreen II 0800‐*LUC*‐*proCsRPP13* or with pGreenII 62‐SK‐*CsMYB28* + pGreen II 0800‐*LUC* empty vector as a control. *N. benthamiana* leaves treated with d‐luciferin potassium salt were observed using the Chemiscope 6100 imaging system. (c) Firefly luciferase and renilla luciferase were detected using the Dual‐Luciferase Reporter Gene Assay Kit. Data are expressed as mean ± SD (*n* = 3). Statistical significance was determined using Student's *t* test (*p < 0.05).

We then investigated whether CsMYB28 regulated *CsRPP13* gene expression. After the pGADT7‐*CsMYB28* vector and the pAbAi‐pro*CsRPP13* vector were transformed into the Y1HGold yeast, the yeast one‐hybrid (Y1H) assay indicated that CsMYB28 bound to the *CsRPP13* promoter (Figure 5a). In addition, the dual‐luciferase assay indicated that the fluorescence value of the region that was co‐injected with pGreenII 62‐SK‐*CsMYB28* and pGreen II 0800‐*LUC*‐*proCsRPP13* significantly increased (Figure 5b), and the LUC/REN ratio of the treatment was also significantly increased (Figure 5c). These results indicated that CsMYB28 is a transcriptional activator of the *CsRPP13* gene.

### 2.6 miR828a Can Cleave *CsMYB28*

**FIGURE 6:**
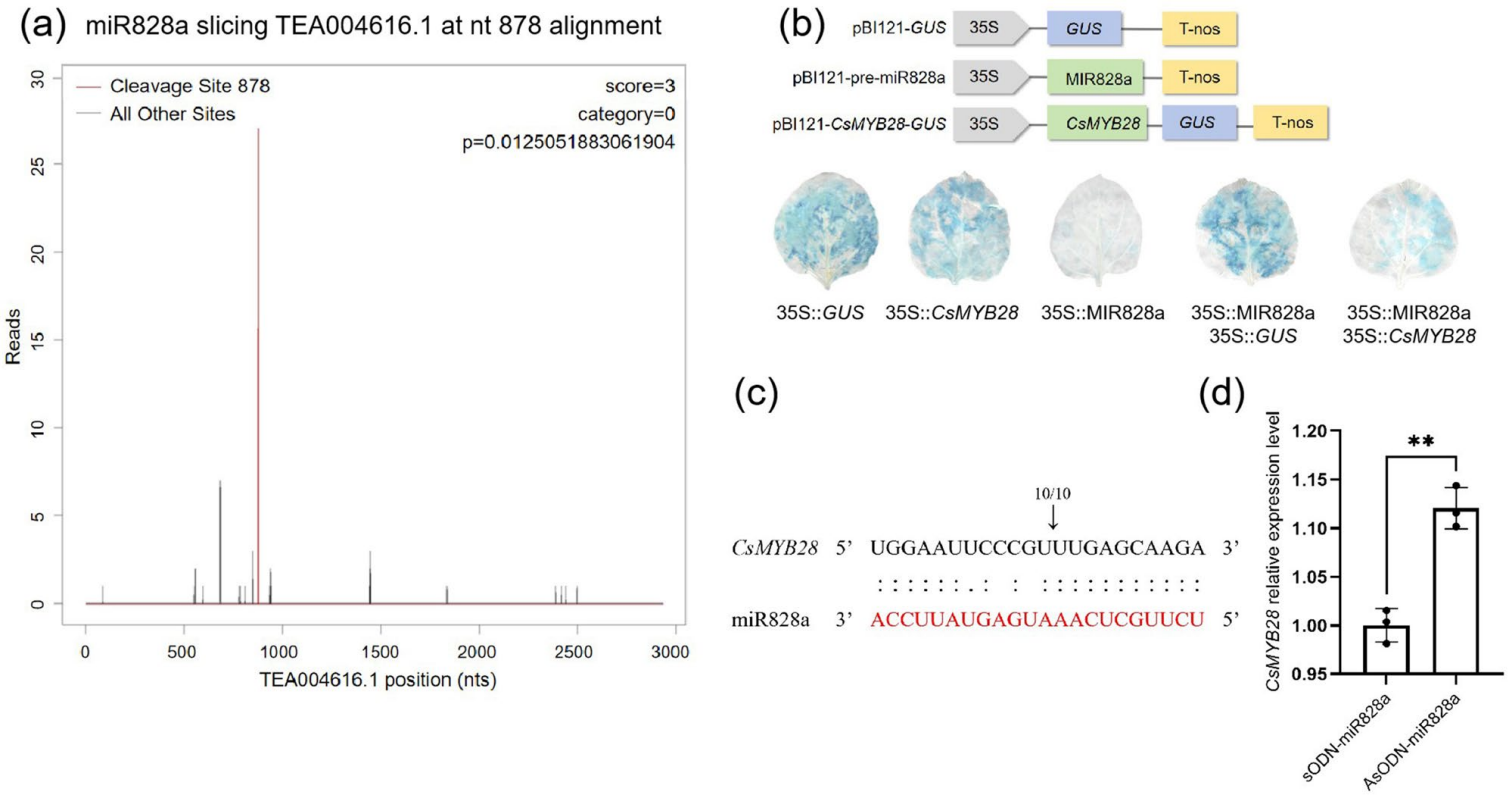
Targeted cleavage of *CsMYB28* mRNA (TEA004616.1) by miR828a. (a) Degradome sequencing revealed that miR828a specifically targeted and cleaved the *CsMYB28* transcript (TEA004616.1) at nucleotide position 878. The red line represents the cleavage site, while the black line illustrates all other sites. (b) β‐glucuronidase (GUS) staining was used to show the relationships between miR828a and *CsMYB28*. 35S:*GUS* was used as the positive controls; 35S:miR828a was used as negative control. (c) 5′ RNA ligase‐mediated‐rapid amplification of cDNA ends (5′ RLM‐RACE) assay validated the relationship between *CsMYB28* and miR828a. (d) The relative expression level of *CsMYB28* in antisense oligonucleotide (AsODN)‐miR828a‐treated and sense oligonucleotide (sODN)‐miR828a‐treated leaves, using reverse transcription‐quantitative PCR. Data are expressed as mean ± SD (*n* = 3). Statistical significance was determined using Student's *t* test (**p < 0.01).

Degradome sequencing revealed that miR828a cleaved *CsMYB28* mRNA at nucleotide 878 (Figure 6a). To confirm that miR828a could cleave the *CsMYB28* mRNA in plants, histochemical β‐glucuronidase (GUS) staining indicated that the staining density in the leaves injected with 35S:*GUS*, 35S:*CsMYB28*, and co‐injected with 35S:miR828a and 35S:*GUS* was significantly deeper due to the expression of the *GUS* gene. Nevertheless, the staining density of the area in leaves resulting from the co‐injection of 35S:miR828a and 35S:*CsMYB28* was relatively light due to the cleavage effect of miR828a on *CsMYB28* mRNA. In addition, the staining density of the area in leaves injected with 35S:miR828a was also relatively light due to the leaves lacking 35S:*GUS* (Figure 6b). The RNA ligase‐mediated rapid amplification of cDNA ends (5′ RLM‐RACE) assay showed that miR828a cleaved *CsMYB28* mRNA at exactly the same location as the predicted binding site of miR828a to *CsMYB28* (Figure 6c). Additionally, RT‐qPCR indicated that silencing by miR828a increased the expression level of *CsMYB28*, suggesting that there was a targeted cleavage relationship between miR828a and *CsMYB28* (Figure 6d). All results indicated that miR828a could directly cleave *CsMYB28* and thereby negatively regulate its expression.

### 2.7 miR828a Negatively Regulates Plant Disease Resistance

**FIGURE 7:**
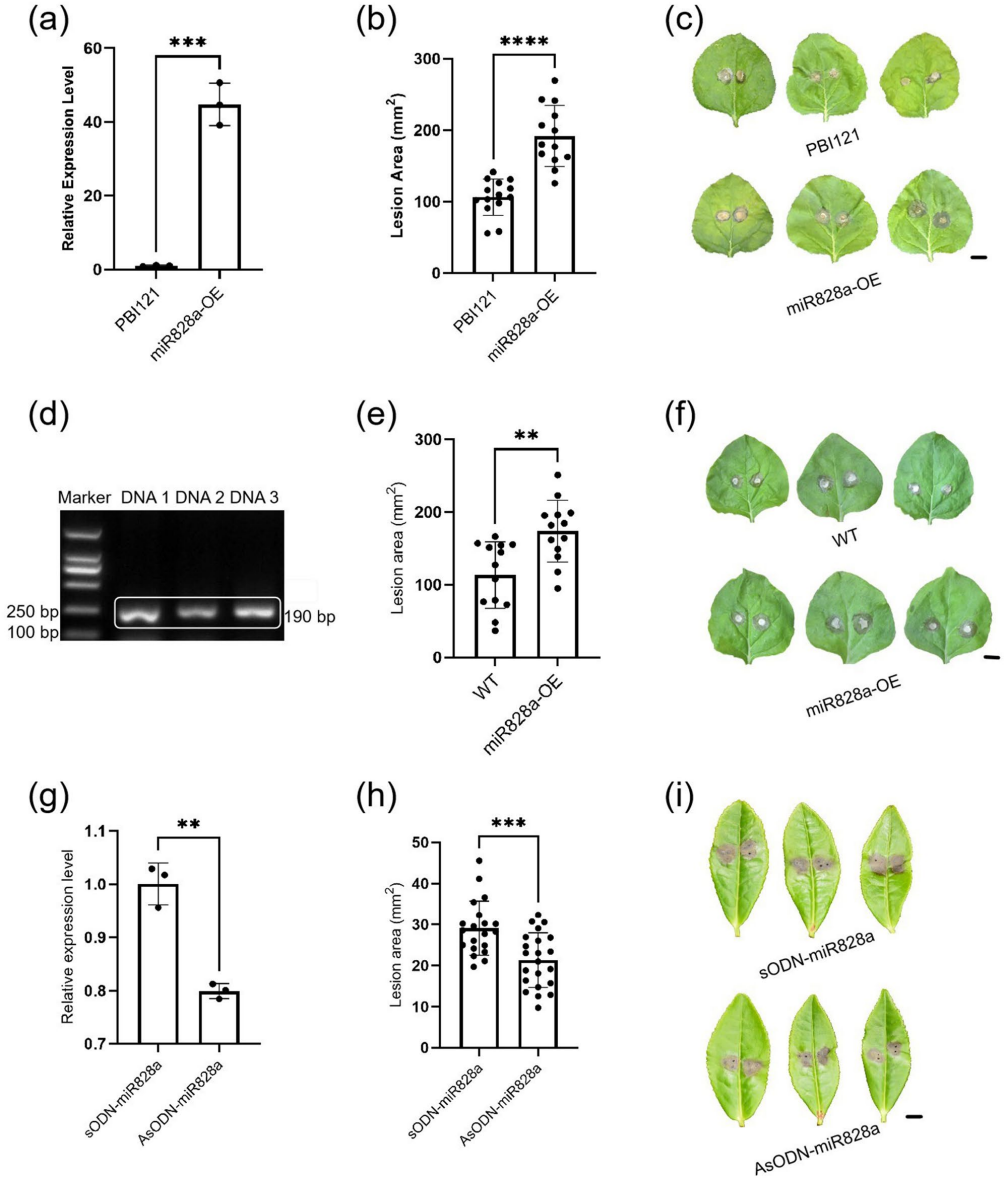
Effects of miR828a on disease resistance. (a) Relative expression levels of miR828a between pBI121 treatment and miR828a‐overexpressing (miR828a‐OE) treatment in *Nicotiana benthamiana* leaves 2 days after injection, as determined by reverse transcription‐quantitative PCR (RT‐qPCR). (b) Lesion areas on *N. benthamiana* leaves 2 days post‐transfection with either pBI121 empty vector or miR828a‐OE, followed by 40 h post‐inoculation with hyphal plugs of *Botrytis cinerea*. (c) Disease symptoms in leaves transfected with pBI121 or miR828a‐OE were observed 40 h post‐inoculation with hyphal plugs of *B. cinerea* . (d) miR828a‐OE transgenic *N. benthamiana* leaves were verified by PCR using specific primers. (e) Lesion areas on wild‐type *N. benthamiana* leaves and miR828a‐OE transgenic *N. benthamiana* leaves were observed 40 h post‐inoculation with *B. cinerea* hyphae. (f) Virulence of *B. cinerea* hyphae on miR828a‐OE transgenic *N. benthamiana* leaves, with wild‐type *N. benthamiana* as a control, was assessed based on lesion size 40 h post‐inoculation. (g) The relative expression level of miR828a in antisense oligonucleotide (AsODN)‐treated and sense oligonucleotide (sODN)‐treated leaves was determined using RT‐qPCR. (h) The lesion areas on AsODN‐miR828a‐treated and sODN‐miR828a‐treated leaves were observed 1 day post‐inoculation with *Lasiodiplodia theobromae* hyphae. (i) The disease symptoms of AsODN‐miR828a‐treated and sODN‐miR828a‐treated tea leaves 1 day after inoculation with *L*. *theobromae* hyphae. All scale bars = 1 cm. Data are expressed as mean ±  SD (*n*  ≥ 3). Statistical significance was determined using Student's *t* test (**p < 0.01, ***p < 0.001, ****p < 0.0001).

To study the role of miR828a in the disease resistance of the tea plant, miR828a was transiently overexpressed in the leaves of *N. benthamiana*; the expression level of miR828a was confirmed by RT‐qPCR (Figure 7a). The leaves with transiently overexpressed miR828a were inoculated with hyphal plugs of *B. cinerea* , and it was found that lesion areas in the transiently overexpressed treatment were significantly larger than those in the control (Figure 7b,c). In addition, the 35S:miR828a vector was constructed and stably overexpressed in *N. benthamiana* plants (Figure 7d). Virulence assays indicated that the transgenic plants produced larger lesions than the control plants after inoculation with *B. cinerea* (Figure 7e,f). To further verify the effect of miR828a on disease resistance, the AsODN method was used to silence the expression of miR828a in tea leaves. RT‐qPCR showed that the expression of miR828a was significantly suppressed, reaching 80% of the control level (Figure 7g). After tea leaves were inoculated with *L. theobromae*, it was found that the area of lesions on tea leaves with silenced miR828a expression as a result of AsODN was significantly smaller than the lesions on non‐silenced leaves treated with sODN (Figure 7h,i). This result indicated that miR828a negatively regulated the defence response of the tea plant. In conclusion, both overexpression and silencing of miR828a indicated that miR828a regulates plant disease resistance.

## 3 Discussion

Plant proteins with transcriptional activation functions play a central role in the plant defence process because they bind to the promoter of target genes and initiate the transcription of downstream regulatory genes (Liu et al. 2005). MYBs, one of the most widely distributed families of TFs in plants, play key roles in plant growth and development, and response to biotic and abiotic stresses (Dubos et al. 2010; Wilkins et al. 2009). Some studies have indicated that MYBs have highly conserved N‐terminal DNA‐binding structural domain repeats (Rs), a more variable C‐terminal region that is responsible for regulatory functions and is involved in plant development and response to stress by binding to MYB *cis*‐elements in the promoters of target genes (Millard et al. 2019; Wang, Niu, and Zheng 2021). *MYB* genes play a role in stress tolerance, and there are *GmMYB* genes in soybeans ( *Glycine max* ), the expression of which changes in response to exposure to abscisic acid (ABA), salt, drought, or cold stress. Specifically, soybean *GmMYB76* and *GmMYB117* confer tolerance to salt and freezing stress (Liao et al. 2008). MYBs are also involved in regulating the synthesis of anthocyanin secondary metabolites (Ma et al. 2024). Studies have revealed that *MYB28* in Chinese kale ( *Brassica oleracea* var. *alboglabra*) plays an important role in the control of aliphatic thioglucoside biosynthesis and has the potential to alter aliphatic glucosinolate concentration in plants at the gene level (Augustine et al. 2013; Yin et al. 2017). Studies on *Arabidopsis thaliana* revealed that *MYB28* plays an important role in ABA signalling during seed germination and early seedling growth (Yu et al. 2016). There have been relatively few studies on the function of MYB28 in regulating genes conferring disease resistance. *SlMYB28* was involved in the regulation of tomato yellow leaf curl virus infection in tomato (Li et al. 2018). In the present study, we found for the first time that *CsMYB28* is an important TF involved in disease resistance in tea bushes. In our study, resistance to pathogenic fungi was enhanced by transient overexpression of *CsMYB28* in *N. benthamiana* leaves and in transgenic *N. benthamiana* plants (Figure 3a–f), and susceptibility to pathogen infection was enhanced by silencing of *CsMYB28* expression in the tea plant by AsODN (Figure 3g–i).

In the current study, *CsMYB28* was confirmed to have transcriptional activation activity by use of the TF self‐activation assay (Figure 1a). The *CsRPP13* gene was selected from the disease‐resistance pathway based on results from the DAP‐seq data (Table S2) and was then used to confirm the activity of transcriptional activation (Figure 5a–c). Some previous studies had indicated that *RPP13* plays an important role in the resistance of plants to pathogen infection, particularly in pathogen recognition and defence mechanism activation (Li et al. 2024). For instance, 21 *RPP13‐like* genes were identified in barley through bioinformatics, using the search criterion of the nucleotide‐binding site leucine‐rich repeat (NBS‐LRR) protein family, which represents most disease‐resistance genes, including the largest number of disease‐resistance genes against downy mildew caused by *Hyaloperonospora parasitica* (Cheng et al. 2018). In *Arabidopsis*, the *AtRPP13* locus contains a single gene or a tightly linked set of genes, which is capable of recognising multiple downy mildew isolates, promoting the disease response of the plant host (Bittner‐Eddy et al. 1999, 2000). The *TaRPP13* gene of wheat ( *Triticum aestivum* ) is involved in the defence response to powdery mildew and plays a positive role in wheat powdery mildew interactions (Liu et al. 2020). In addition, *ZmRPP13* of maize ( *Zea mays* ) encodes a novel adenylate cyclase (AC), which is also involved in abscisic acid‐mediated heat tolerance (Yang et al. 2021). These studies illustrated that *RPP13* exhibits a wide range of biological activities and is involved in both biotic and abiotic stress responses in many plants. However, *CsRPP13* in the tea plant was less studied. In the present study, we showed that *CsMYB28* could bind to the promoter of the *CsRPP13* disease‐resistance gene and initiate its transcription (Figure 5a–c). All of the above results indicated that *CsMYB28* is involved in plant disease resistance by directly binding to the *CsRPP13* promoter to activate its transcription.

Previously, miRNAs had been shown to mediate gene regulatory pathways, participate in the negative regulation of gene expression, and play key roles in developmental and metabolic pathways (Saliminejad et al. 2019). In studies of plants such as *A. thaliana* , lotus, apple, and grape, a number of miRNAs could target and cleave the mRNAs of MYB TFs, which were known to be the regulators of the phenylpropanoid metabolic pathway (Tirumalai et al. 2019). Furthermore, several miRNAs respond to various pathogens in a positive or negative manner in different species (Li et al. 2019). In purple potato, miR828a regulates anthocyanin biosynthesis (Wu et al. 2022) and plays active roles in pitaya fruit colouration and accumulation of betalains, a pigment family unrelated to anthocyanins (Chen et al. 2020). In the tea bush, miR828a negatively regulates the biosynthesis of theobromine (Jin et al. 2024). In tea leaves infected with *Colletotrichum gloeosporioides*, the expression profile of miR828a shows a negative correlation with that of its target gene *MYB75*, which is known to be a positive regulator of abiotic stress responses, implying that miR828a might play a negative role in the abiotic stress response in tea (Jeyaraj et al. 2019).

However, the role of miR828a in the disease‐resistance mechanism of the tea plant has not been studied. In the current study, we confirmed that miR828a exerted a negative regulation of disease resistance by targeting the cleavage of *CsMYB28* mRNA. The targeting by miR828a of the cleavage of *CsMYB28* mRNA was confirmed by GUS histochemical staining (Figure 6b). It was also confirmed by degradome sequencing and by a modified 5′ RLM‐RACE assay that miR828a cleaved *CsMYB28* mRNA at nucleotide position 878 (Figure 6a,c). In addition, when miR828a was silenced in tea leaves by the AsODN method, *CsMYB28* abundance was significantly increased, a finding that was confirmed by the lower inhibition of *CsMYB28* expression by miR828a (Figure 6d). The transient overexpression as well as the stable overexpression of miR828a in *N. benthamiana* leaves showed that the plants exhibited significantly larger disease lesions when challenged by *B. cinerea* , which indicated that miR828a expression could increase plant susceptibility to the pathogen (Figure 7a–f). Meanwhile, silencing of miR828a in tea leaves by AsODN caused a significant reduction in the size of tea leaf lesions, which indicated that miR828a could decrease plant resistance to infection (Figure 7g–i). All of the above experimental results indicated that miR828a targets the cleavage of the *CsMYB28* mRNA, thus achieving the negative regulation of plant disease resistance.

When a plant is challenged by pathogens, it is able to activate defence responses, thereby inhibiting infection or colonisation by the pathogens (Hammond‐Kosack and Jones 1997). The plant host can effectively regulate its defence responses against pathogens at the RNA and/or protein level (Yang et al. 2020). The miRNA/mRNA‐mediated gene regulation model has been reported to involve many functions in plants other than tea. For example, *VvMYB114*, mediated by miR828, negatively regulates trichome development of *A. thaliana* (Chen et al. 2021), while miR828 and miR858 regulate *VvMYB114* to promote the expression of the downstream genes associated with anthocyanin and flavonol accumulation in grapes (Tirumalai et al. 2019); mdm‐miR858 negatively regulates proanthocyanidin accumulation by targeting *MdMYB9/11/12* in apple peel (Zhang et al. 2022). In the downstream gene regulation module, *MYB28* positively regulates *CYP79F1, CYP79F2*, and *CYP83A1*, which are involved in aliphatic thioglucoside biosynthesis (Yin et al. 2017). In the tea plant, the miR828a–*CsMYB114*–*CsTbS* module negatively regulates the biosynthesis of theobromine in *Camellia sinensis* (Jin et al. 2024). Previous work by our research group found that the lncRNA81246–miR164d–*CsNAC1*–*CsEXLB1* module regulates resistance against tea leaf spot caused by *L. theobromae* (Guo et al. 2025).

In the present study, we found that *CsMYB28* played an important role in increasing plant resistance. For instance, the abundance of *CsMYB28* mRNA was disturbed by miR828a action (Figure 6a–d), leading to the suppression of plant resistance (Figure 7a–i). Our results demonstrated that miR828a affected plant defence against phytopathogens by interfering with the *CsMYB28*‐mediated disease‐resistance pathway. We also found that CsMYB28 bound to the promoter of *CsRPP13*, promoting its transcription and thereby enhancing plant disease resistance. Based on these findings, we proposed a new defence module of miR828a–*CsMYB28*–*CsRPP13* in tea leaves following challenge by *L. theobromae*, representing a new module in the regulation of defence in tea, which could provide valuable data for breeding disease‐resistant tea plants. In addition, no literature reported the relationship between the biomolecules in the two regulatory modules, lncRNA81246–miR164d–*CsNAC1*–*CsEXLB1* and miR828a–*CsMYB28*–*CsRPP13*. It remains unclear whether these two regulatory modules are interconnected. Given the limited research on *L. theobromae* and the diseases caused by this pathogen, we plan to further investigate the response mechanisms of *L. theobromae* following its infection of tea plants in future studies.

## 4 Experimental Procedures

### 4.1 Plant Growth and Pathogen Inoculation

Potted 5‐year‐old tea plants (*C. sinensis* ‘Fuding‐dabaicha’) were grown in a greenhouse maintained at 25°C during the day and 20°C at night, with photoperiod cycles of 14 h light and 10 h darkness and a relative humidity of 70%–80%. *N. benthamiana* plants were grown in 10 cm pots filled with a mixture of 60% vermiculite and 40% meadow soil, kept at 25°C during the day and 20°C at night, with a cycle of 14 h of light and 10 h of darkness. The pathogens *L. theobromae* CGMCC 3.20151 and *B. cinerea* CGMCC3.20932 were grown on potato dextrose agar (PDA), and virulence was determined using hyphal plugs on PDA in accordance with the methods of our research group (Guo et al. 2025). *L. theobromae* was inoculated onto tea leaves for 24 h, while *B. cinerea* was inoculated onto *N. benthamiana* leaves for 40 h. Each experiment contained at least three biological replicates per treatment. The experiment was independently conducted three times, and similar results were obtained.

### 4.2 Bioinformatic Analyses of CsMYB28

The protein sequences were downloaded from the National Center for Biotechnology Information (https://blast.ncbi.nlm.nih.gov/Blast.cgi). Briefly, ClustalW software (https://www.genome.jp/tools‐bin/clustalw) was used for multiple sequence alignments. A phylogenetic tree was constructed using the MEGA X (https://www.megasoftware.net/) proximity method (neighbour‐joining) with 1000 bootstrap replications. Protein sequences were compared and domains were analysed using GeneDoc software (http://nrbsc.org/gfx/genedoc/index.html).

### 4.3 Total RNA Extraction and RT‐qPCR

Total RNA was extracted using the TransZol Up Plus RNA Kit (TransGen). Synthesis of first‐strand cDNA was performed using an EasyScript One‐Step gDNA Removal and cDNA Synthesis SuperMix Kit (TransGen). RT‐qPCR was performed using Premix Ex Taq (TaKaRa). Gene expression levels were analysed using gene‐specific primers (Table S1). Relative gene expression was calculated using the 2^−ΔΔ*C*t^ method (Livak and Schmittgen 2001).

### 4.4 Subcellular Localisation of CsMYB28

Homologous recombination primers for the coding sequence (CDS)‐free terminator sequence of *CsMYB28* were designed using the online tool available at https://crm.vazyme.com/cetool/singlefragment.html (Table S1). The CDS‐free terminator sequence was subsequently ligated into the pCAMBIA2300::*EGFP* vector via the BamHI and SalI restriction sites, generating a fusion vector, 35S CaMV:*CsMYB28*‐*EGFP*. The fusion vector of 35SCaMV*:CsMYB28‐EGFP* and the negative control pCAMBIA2300::*EGFP* were transfected into *N. benthamiana* leaves by infiltration of *Agrobacterium tumefaciens* GV3101. After 2 days, the leaves were imaged using a confocal microscope (LSM 900; Zeiss).

### 4.5 Transactivation Activity Assay

The coding sequence of *CsMYB28* was inserted into pGBKT7. Subsequently, the pGBKT7*::CsMYB28* and pGBKT7 vectors were transformed into yeast strain AH109. Finally, following positive PCR detection, individual colonies of positive clones were selected and cultured in the synthetic defined (SD) selective media SD/−Trp, SD/−Trp‐His, SD/−Trp‐His/X‐*α*‐gal, SD/−Trp‐His‐Ade, and SD/−Trp‐His‐Ade/X‐*α*‐gal in the dark at 30°C for 3 days. The primers used are listed in Table S1.

### 4.6 Spatiotemporal *CsMYB28* Expression

The first, second, and third leaves of the detached tea plant were inoculated with the hyphal plugs of *L. theobromae* on PDA for 12, 24, 36, and 48 h. Total RNA was extracted from the samples, and the abundance of *CsMYB28* mRNA in the samples was quantified using RT‐qPCR to measure expression. The primers used are listed in Table S1.

### 4.7 Transient Gene Expression in *N. benthamiana* and Generation of Transgenic Plants

The coding sequences of *CsMYB28* and miR828a precursor sequences were individually inserted into pBI121 using XbaI and SacI and transferred into *A. tumefaciens* GV3101 (Table S1). The empty pBI121 vector was used as the control. Leaves were harvested 2 days after infiltration for experiments. After confirming successful overexpression, *B. cinerea* was used in a 40‐h virulence assay.

The generation of transgenic *N. benthamiana* was performed as described previously, with minor modifications (Khare et al. 2010). Explants were prepared from excised cotyledons obtained from 2‐month‐old *N. benthamiana* seedlings using the leaf disc method and then inoculated by infiltration with *A. tumefaciens* GV3101 containing the vectors pBI121‐*CsMYB28* and pBI121‐*MIR828a*. The positive plants were selected on Murashige and Skoog (MS) medium containing 50 mg/L kanamycin. DNA was extracted, and PCR was used to verify the transgenic plants using specific primers (Table S1). Wild‐type *N. benthamiana* was used as a control in the virulence experiments.

### 4.8 Antisense Oligonucleotide Silencing

The Soligo online tool (https://sfold.wadsworth.org/cgi‐bin/soligo.pl) was used to select the appropriate antisense oligonucleotides. The AsODN procedure was performed as described previously (Li et al. 2022). In brief, tea shoots, with one apical bud and two leaves, were detached and then placed in Eppendorf tubes containing 1 mL of 20 μM AsODN‐*CsMYB28* or AsODN‐miR828a for 24 h. The second (i.e., the youngest) leaf was inoculated with two PDA plugs containing *L. theobromae* hyphae for 24 h (Guo et al.  2025). The corresponding sense oligonucleotides (sODN) acted as controls. The primers used are listed in Table S1.

### 4.9 DNA Affinity Purification Sequencing (DAP‐Seq) Analysis

DAP‐seq assay was performed as the method described previously (Orduña et al. 2022). Total DNA was extracted from the tea leaves and then fragmented to a size of 200 bp. To generate the adaptor, adenosine monophosphate was ligated to the end of the fragmented DNA. The coding sequence of *CsMYB28* was ligated into a p30a‐HaloTag vector and then expressed in TnT SP6 High‐Yield Germ Master Mix. The MANAGE HaloTag Beads (Promega Biotech) were used to purify the fusion protein. After the fusion protein bound to the DNA, the unbound DNA was washed away, and the bound DNA was used to generate sequencing libraries on an Illumina HiSeq. Each peak was annotated using the genome database (TPIA; http://tpia.teaplant.org), and the recognition motifs of CsMYB28 were identified. Gene Ontology (GO) enrichment analysis was performed using the software GOATOOLS (https://github.com/tanghaibao/GOatools), while Kyoto Encyclopedia of Genes and Genomes (KEGG) enrichment analysis was performed using KOBAS (http://kobas.cbi.pku.edu.cn/kobas3/?t=1). A scatter plot for the DEGs was generated. Fisher's exact probability test was used for statistical analysis.

### 4.10 Yeast One‐Hybrid Assay

The assay was conducted as described previously (Huai et al. 2022). Briefly, the coding sequence of *CsMYB28* was inserted into the pGADT7 vector. The promoter of *CsRPP13*, containing the *CsMYB28 cis*‐regulatory element, was ligated into the pAbAi vector. The plasmid was extracted and digested using BstBI, and then transformed into the yeast strain Y1H Gold, which was then plated onto SD/−Ura selective medium (Clontech). The construct pGADT7‐*CsMYB28* was transformed into yeast prepared by pAbAi‐pro*CsRPP13* and plated on SD/−Leu selective medium containing the selected aureobasidin A (AbA) concentration containing 0, 50, 150, 300, and 500 ng/mL. *p53*‐pAbAi was used as the positive control. The primers used are listed in Table S1.

### 4.11 Dual‐Luciferase Assay

The dual‐luciferase assay was performed as described previously, with minor modifications (Du et al. 2020; Zhang et al. 2024). *CsMYB28* was linked into the pGreen II 62‐SK vector to generate the pGreen II 62‐SK‐*CsMYB28* construct, and the promoter sequence of *CsRPP13* was linked into the pGreen II 0800‐*LUC* vector to generate the pGreen II 0800‐*LUC*‐pro*CsRPP13* construct. Every construct was transformed into *A. tumefaciens* GV3101 (pSoup). Different regions of the same *N. benthamiana* leaf were injected with pGreen II 0800‐*LUC*‐*proCsRPP13* + pGreen II 62‐SK‐*CsMYB28*, and pGreen II 62‐SK empty vector + pGreen II 0800‐LUC‐pro*CsRPP13* or pGreen II 62‐SK‐*CsMYB28* + pGreen II 0800‐*LUC* empty vector and incubated for 2 days. d‐Luciferin potassium salt (Biyuntian) was dissolved in sterile phosphate‐buffered saline (free of Mg^2+^ and Ca^2+^) (pH 7.4, 0.01 M) to prepare a 15 mg/mL solution. The solution was filtered through a 0.2‐μm pore‐size filter and then diluted with sterile water to 1:200 to achieve a final concentration of 150 μg/mL. After incubating at 37°C for 5 min, the leaves were immediately analysed using the ChemiScope Capture image acquisition software and an imaging system (ChemiScope 6100; Shanghai Qinxiang Scientific Instrument Co. Ltd.). The activities of firefly luciferase and renilla luciferase were detected using the Dual‐Luciferase Reporter Gene Assay Kit (Yeasen Biotechnology) on the Multi‐Function Measuring Instrument (Feyond‐A300, Allsheng).

### 4.12 Degradome Sequencing

Degradome sequencing was performed as described previously, with minor modifications (Lin et al. 2016). Total RNA from control (CK, uninfected leaves) and treated (infected) leaves was used to construct two degradome libraries (CK treatment and infected treatment). ACGT101‐DEG (LC Sciences, http://www.lcsciences.com/) and CleaveLand 3.0 software packages (https://sites.psu.edu/axtell/software/cleaveland3/) were used to detect possible miRNA targets. All the targets were distinguished based on their abundance.

### 4.13 Histochemical GUS Staining

The coding sequences of *CsMYB28* and miR828a precursor were independently inserted into the pBI121 vector and transferred into receptor cells of *A. tumefaciens* GV3101. Two days after transformation, tissues were stained to visualise GUS activity as previously described (Jefferson et al. 1987). The pBI121‐*GUS* vector was used as a control. Primers are listed in Table S1.

### 4.14 5′ RLM‐RACE

5′ RLM‐RACE was performed according to the previously described method with minor modifications (Wang, Liu, et al. 2021; Guo et al. 2025). Total RNA was extracted from tea leaves using the TransZol UP Plus RNA Kit (TransGen), following the manufacturer's instructions. The 5′ RNA adapter was ligated using T4 RNA ligase. cDNA templates were amplified by PCR using the 5′ outer primer and the 3′ gene‐specific primer. The PCR product was then amplified by nested PCR with the 5′ inner primer and inner‐1 or inner‐2 primers (Table S1). The PCR products were purified from agarose gel and ligated into the T‐vector for sequencing to analyse the cleavage site.

### 4.15 Statistical Analysis

Statistical analysis was performed by Prism 9.0 (GraphPad). For multiple comparisons (of more than two samples), *p‐*values were calculated by using one‐way ANOVA and Dunnett's multiple comparison test. The paired comparison used Student's *t* test. All data were presented as mean ± SD.

## Conflicts of Interest

The authors declare no conflicts of interest.

## Supporting information


**Figure S1.** A phylogenetic analysis of *CsMYB28*.

**Figure S2.** Distribution of peak length by DAP‐seq.

**Figure S3.** The average abundance of reads near CsMYB28‐binding regions within the 10‐kb region upstream and downstream of the transcription start site (TSS) for CsMYB28‐binding regions.

**Table S1.** The list of primer and oligonucleotide sequence for the genes in this study.

**Table S2.** DNA affinity purification‐sequencing data.

## Data Availability

The data that support the findings of this study are provided in the Supporting Information files.

## References

[mpp70069-bib-0001] Augustine, R. , M. Majee , J. Gershenzon , and N. C. Bisht . 2013. “Four Genes Encoding MYB28, a Major Transcriptional Regulator of the Aliphatic Glucosinolate Pathway, Are Differentially Expressed in the Allopolyploid *Brassica juncea* .” Journal of Experimental Botany 64: 4907–4921. 24043856 10.1093/jxb/ert280 PMC3830477

[mpp70069-bib-0002] Aukerman, M. J. , and H. Sakai . 2003. “Regulation of Flowering Time and Floral Organ Identity by a MicroRNA and Its *APETALA2*‐Like Target Genes.” Plant Cell 15: 2730–2741. 14555699 10.1105/tpc.016238 PMC280575

[mpp70069-bib-0003] Bittner‐Eddy, P. , C. Can , N. Gunn , et al. 1999. “Genetic and Physical Mapping of the *RPP13* Locus, in *Arabidopsis*, Responsible for Specific Recognition of Several *Peronospora parasitica* (Downy Mildew) Isolates.” Molecular Plant–Microbe Interactions 12: 792–802. 10494631 10.1094/MPMI.1999.12.9.792

[mpp70069-bib-0004] Bittner‐Eddy, P. , I. R. Crute , E. B. Holub , and J. L. Beynon . 2000. “ *RPP13* Is a Simple Locus in *Arabidopsis thaliana* for Alleles That Specify Downy Mildew Resistance to Different Avirulence Determinants in *Peronospora parasitica* .” Plant Journal 21: 177–188. 10.1046/j.1365-313x.2000.00664.x 10743658

[mpp70069-bib-0005] Chen, C. , F. Xie , Q. Hua , et al. 2020. “Integrated sRNAome and RNA‐Seq Analysis Reveals miRNA Effects on Betalain Biosynthesis in Pitaya.” BMC Plant Biology 20: 437. 32962650 10.1186/s12870-020-02622-x PMC7510087

[mpp70069-bib-0006] Chen, Q. , J. Wang , P. Danzeng , C. Danzeng , S. Song , and L. Wang . 2021. “ *VvMYB114* Mediated by miR828 Negatively Regulates Trichome Development of *Arabidopsis* .” Plant Science 309: 110936. 34134843 10.1016/j.plantsci.2021.110936

[mpp70069-bib-0007] Cheng, J. , H. Fan , L. Li , B. Hu , H. Liu , and A. Liu . 2018. “Genome‐Wide Identification and Expression Analyses of *RPP13*‐Like Genes in Barley.” BioChip Journal 12: 102–113.

[mpp70069-bib-0008] David, P. B. 2004. “MicroRNAs: Genomics, Biogenesis, Mechanism, and Function.” Cell 116: 281–297. 14744438 10.1016/s0092-8674(04)00045-5

[mpp70069-bib-0009] Du, S. S. , L. Li , L. Li , et al. 2020. “Photoexcited Cryptochrome2 Interacts Directly With TOE1 and TOE2 in Flowering Regulation.” Plant Physiology 184: 487–505. 32661061 10.1104/pp.20.00486 PMC7479908

[mpp70069-bib-0010] Dubos, C. , R. Stracke , E. Grotewold , B. Weisshaar , C. Martin , and L. Lepiniec . 2010. “MYB Transcription Factors in *Arabidopsis* .” Trends in Plant Science 15: 573–581. 20674465 10.1016/j.tplants.2010.06.005

[mpp70069-bib-0011] Feng, H. , X. Duan , Q. Zhang , et al. 2014. “The Target Gene of Tae‐miR164, a Novel NAC Transcription Factor From the NAM Subfamily, Negatively Regulates Resistance of Wheat to Stripe Rust.” Molecular Plant Pathology 15: 284–296. 24128392 10.1111/mpp.12089 PMC6638668

[mpp70069-bib-0012] Guo, D. , D. Li , F. Liu , et al. 2025. “LncRNA81246 Regulates Resistance Against Tea Leaf Spot by Interrupting the miR164d‐Mediated Degradation of NAC1.” Plant Journal 121: e17173. 10.1111/tpj.17173 PMC11711933 39590921

[mpp70069-bib-0013] Guo, D. , Z. Xia , X. Jiang , et al. 2022. “Sequencing and Functional Annotation of Competing Endogenous RNAs and MicroRNAs in Tea Leaves During Infection by *Lasiodiplodia theobromae* .” PhytoFrontiers 2: 307–313.

[mpp70069-bib-0014] Guo, H. , Q. Xie , J. Fei , and N. Chua . 2005. “MicroRNA Directs mRNA Cleavage of the Transcription Factor *NAC1* to Downregulate Auxin Signals for *Arabidopsis* Lateral Root Development.” Plant Cell 17: 1376–1386. 15829603 10.1105/tpc.105.030841 PMC1091761

[mpp70069-bib-0015] Hammond‐Kosack, K. E. , and J. D. G. Jones . 1997. “Plant Disease Resistance Genes.” Annual Review of Plant Biology 48: 575–607. 10.1146/annurev.arplant.48.1.575 15012275

[mpp70069-bib-0016] Huai, B. , P. Yuan , X. Ma , et al. 2022. “Sugar Transporter TaSTP3 Activation by TaWRKY19/61/82 Enhances Stripe Rust Susceptibility in Wheat.” New Phytologist 236: 266–282. 35729085 10.1111/nph.18331

[mpp70069-bib-0017] Jefferson, R. A. , T. A. Kavanagh , and M. W. Bevan . 1987. “GUS Fusions: β‐Glucuronidase as a Sensitive and Versatile Gene Fusion Marker in Higher Plants.” EMBO Journal 6: 3901–3907. 3327686 10.1002/j.1460-2075.1987.tb02730.x PMC553867

[mpp70069-bib-0018] Jeyaraj, A. , X. Wang , S. Wang , et al. 2019. “Identification of Regulatory Networks of MicroRNAs and Their Targets in Response to *Colletotrichum gloeosporioides* in Tea Plant (*Camellia sinensis* L.).” Frontiers in Plant Science 10: 1096. 31572415 10.3389/fpls.2019.01096 PMC6751461

[mpp70069-bib-0019] Jin, Q. , Z. Wang , D. Sandhu , et al. 2024. “miR828a‐CsMYB114 Module Negatively Regulates the Biosynthesis of Theobromine in *Camellia sinensis* .” Journal of Agricultural and Food Chemistry 8: 4464–4475. 10.1021/acs.jafc.3c07736 38376143

[mpp70069-bib-0020] Khare, N. , D. Goyary , N. K. Singh , et al. 2010. “Transgenic Tomato cv. Pusa Uphar Expressing a Bacterial Mannitol‐1‐Phosphate Dehydrogenase Gene Confers Abiotic Stress Tolerance.” Plant Cell Tissue and Organ Culture 103: 267–277.

[mpp70069-bib-0021] Li, C. , B. Yuan , C. Zhang , et al. 2024. “Revealing Key Genes and Pathways in Potato Scab Disease Resistance Through Transcriptome Analysis.” Agronomy 14: 291.

[mpp70069-bib-0022] Li, D. , S. Jiang , X. Wen , et al. 2021. “Sequencing and Functional Annotation of mRNAs and lncRNAs From Tea (*Camellia sinensis*) Leaves During Infection by the Fungal Pathogen *Lasiodiplodia theobromae* .” PhytoFrontiers 1: 364–367.

[mpp70069-bib-0023] Li, T. , X. Zhang , Y. Huang , Z. Xu , F. Wang , and A. Xiong . 2018. “An R2R3‐MYB Transcription Factor, *SlMYB28*, Involved in the Regulation of TYLCV Infection in Tomato.” Scientia Horticulturae 237: 192–200.

[mpp70069-bib-0024] Li, X. , L. Jin , Z. Chen , and B. Song . 2022. “Application and Development of Green Preventive and Control Technologies in Guizhou Tea Plantations.” Frontiers of Agricultural Science and Engineering 9: 75–81.

[mpp70069-bib-0025] Li, Y. , J. M. J. Jeyakumar , Q. Feng , et al. 2019. “The Roles of Rice MicroRNAs in Rice–*Magnaporthe oryzae* Interaction.” Phytopathology Research 1: 33.

[mpp70069-bib-0026] Liao, Y. , H. Zou , H. Wang , et al. 2008. “Soybean *GmMYB76*, *GmMYB92*, and *GmMYB177* Genes Confer Stress Tolerance in Transgenic *Arabidopsis* Plants.” Cell Research 18: 1047–1060. 18725908 10.1038/cr.2008.280

[mpp70069-bib-0027] Lin, P. C. , C. W. Lu , B. N. Shen , et al. 2016. “Identification of miRNAs and Their Targets in the Liverwort *Marchantia polymorpha* by Integrating RNA‐Seq and Degradome Analyses.” Plant Cell Physiology 57: 339–358. 26861787 10.1093/pcp/pcw020 PMC4788410

[mpp70069-bib-0028] Liu, H. , S. Jia , D. Shen , et al. 2012. “Four AUXIN RESPONSE FACTOR Genes Downregulated by MicroRNA167 Are Associated With Growth and Development in *Oryza sativa* .” Functional Plant Biology 39: 736–744. 32480824 10.1071/FP12106

[mpp70069-bib-0029] Liu, L. , H. Chen , J. Zhu , L. Tao , and C. Wei . 2022. “miR319a Targeting of *CsTCP10* Plays an Important Role in Defense Against Gray Blight Disease in Tea Plant (*Camellia sinensis*).” Tree Physiology 42: 1450–1462. 35099563 10.1093/treephys/tpac009

[mpp70069-bib-0030] Liu, X. , X. Bai , Q. Qian , et al. 2005. “ *OsWRKY03*, a Rice Transcriptional Activator That Functions in Defense Signaling Pathway Upstream of *OsNPR1* .” Cell Research 15: 593–603. 16117849 10.1038/sj.cr.7290329

[mpp70069-bib-0031] Liu, X. , C. Zhang , L. Zhang , et al. 2020. “ *TaRPP13‐3*, a CC‐NBS‐LRR‐Like Gene Located on Chr 7D, Promotes Disease Resistance to Wheat Powdery Mildew in Brock.” Journal of Phytopathology 168: 688–699.

[mpp70069-bib-0032] Livak, K. J. , and T. D. Schmittgen . 2001. “Analysis of Relative Gene Expression Data Using Real‐Time Quantitative PCR and the 2(−ΔΔC(T)) Method.” Methods 25: 402–408. 11846609 10.1006/meth.2001.1262

[mpp70069-bib-0033] Lu, M. , Y. Zhao , Y. Feng , et al. 2024. “2,4‐Dihydroxybenzoic Acid, a Novel SA Derivative, Controls Plant Immunity via *UGT95B17*‐Mediated Glucosylation: A Case Study in *Camellia sinensis* .” Advanced Science 11: 2307051. 38063804 10.1002/advs.202307051 PMC10870048

[mpp70069-bib-0034] Lv, W. , H. Jiang , Q. Cao , et al. 2024. “A Tau Class Glutathione S‐Transferase in Tea Plant, *CsGSTU45*, Facilitates Tea Plant Susceptibility to *Colletotrichum camelliae* Infection Mediated by Jasmonate Signaling Pathway.” Plant Journal 117: 1356–1376. 10.1111/tpj.16567 38059663

[mpp70069-bib-0035] Ma, R. , W. Huang , Q. Hu , et al. 2024. “Tandemly Duplicated MYB Genes Are Functionally Diverged in the Regulation of Anthocyanin Biosynthesis in Soybean.” Plant Physiology 194: 2549–2563. 38235827 10.1093/plphys/kiae019

[mpp70069-bib-0036] Ma, R. , B. Liu , X. Geng , et al. 2023. “Biological Function and Stress Response Mechanism of MYB Transcription Factor Family Genes.” Journal of Plant Growth Regulation 42: 83–95.

[mpp70069-bib-0037] Millard, P. S. , B. B. Kragelund , and M. Burow . 2019. “R2R3 MYB Transcription Factors ‐ Functions Outside the DNA‐Binding Domain.” Trends in Plant Science 24: 934–946. 31358471 10.1016/j.tplants.2019.07.003

[mpp70069-bib-0038] Orduña, L. , M. Li , D. Navarro‐Payá , et al. 2022. “Direct Regulation of Shikimate, Early Phenylpropanoid, and Stilbenoid Pathways by Subgroup 2 R2R3‐MYBs in Grapevine.” Plant Journal 110: 529–547. 10.1111/tpj.15686 35092714

[mpp70069-bib-0039] Palos, K. , L. Yu , C. E. Railey , A. C. Nelson Dittrich , and A. D. L. Nelson . 2023. “Linking Discoveries, Mechanisms, and Technologies to Develop a Clearer Perspective on Plant Long Noncoding RNAs.” Plant Cell 35: 1762–1786. 36738093 10.1093/plcell/koad027 PMC10226578

[mpp70069-bib-0040] Pandey, A. K. , G. D. Sinniah , A. Babu , and A. Tanti . 2021. “How the Global Tea Industry Copes With Fungal Diseases Challenges and Opportunities.” Plant Disease 105: 1868–1879. 33734810 10.1094/PDIS-09-20-1945-FE

[mpp70069-bib-0041] Saliminejad, K. , H. R. Khorram Khorshid , S. Soleymani Fard , and S. H. Ghaffari . 2019. “An Overview of MicroRNAs: Biology, Functions, Therapeutics, and Analysis Methods.” Journal of Cellular Physiology 234: 5451–5465. 30471116 10.1002/jcp.27486

[mpp70069-bib-0042] Samarfard, S. , A. Ghorbani , T. P. Karbanowicz , et al. 2022. “Regulatory Non‐coding RNA: The Core Defense Mechanism Against Plant Pathogens.” Journal of Biotechnology 359: 82–94. 36174794 10.1016/j.jbiotec.2022.09.014

[mpp70069-bib-0043] Schwab, R. , J. F. Palatnik , M. Riester , et al. 2005. “Specific Effects of MicroRNA on the Plant Transcriptome.” Developmental Cell 8: 517–527. 15809034 10.1016/j.devcel.2005.01.018

[mpp70069-bib-0044] Sun, P. , C. Cheng , Y. Lin , Q. Zhu , J. Lin , and Z. Lai . 2017. “Combined Small RNA and Degradome Sequencing Reveals Complex MicroRNA Regulation of Catechin Biosynthesis in Tea (*Camellia sinensis*).” PLoS One 12: 0171173. 10.1371/journal.pone.0171173 PMC5321428 28225779

[mpp70069-bib-0045] Tirumalai, V. , C. Swetha , A. Nair , A. Pandit , and P. V. Shivaprasad . 2019. “miR828 and miR858 Regulate VvMYB114 to Promote Anthocyanin and Flavonol Accumulation in Grapes.” Journal of Experimental Botany 18: 4775–4792. 10.1093/jxb/erz264 PMC6760283 31145783

[mpp70069-bib-0046] Wang, M. , H. Wu , J. Fang , C. Chu , and X. Wang . 2017. “A Long Noncoding RNA Involved in Rice Reproductive Development by Negatively Regulating Osa‐miR160.” Science Bulletin 62: 470–475. 36659255 10.1016/j.scib.2017.03.013

[mpp70069-bib-0047] Wang, S. , L. Liu , X. Mi , et al. 2021. “Multi‐Omics Analysis to Visualize the Dynamic Roles of Defense Genes in the Response of Tea Plants to Gray Blight.” Plant Journal 106: 862–875. 10.1111/tpj.15203 33595875

[mpp70069-bib-0048] Wang, S. , S. Liu , L. Liu , et al. 2020. “miR477 Targets the Phenylalanine Ammonia‐Lyase Gene and Enhances the Susceptibility of the Tea Plant ( *Camellia sinensis* ) to Disease During *Pseudopestalotiopsis* Species Infection.” Planta 251: 59. 32025888 10.1007/s00425-020-03353-x

[mpp70069-bib-0049] Wang, X. , Y. Niu , and Y. Zheng . 2021. “Multiple Functions of MYB Transcription Factors in Abiotic Stress Responses.” International Journal of Molecular Sciences 22: 6125. 34200125 10.3390/ijms22116125 PMC8201141

[mpp70069-bib-0050] Wang, X. , Q. Yin , S. Jiang , et al. 2019. “First Report of *Didymella bellidis* Causing Tea Leaf Spot in China.” Plant Disease 104: 1254.

[mpp70069-bib-0051] Wilkins, O. , H. Nahal , J. Foong , N. J. Provart , and M. M. Campbell . 2009. “Expansion and Diversification of the *Populus* R2R3‐MYB Family of Transcription Factors.” Plant Physiology 149: 981–993. 19091872 10.1104/pp.108.132795 PMC2633813

[mpp70069-bib-0052] Wu, X. , Y. Ma , J. Wu , et al. 2022. “Identification of MicroRNAs and Their Target Genes Related to the Accumulation of Anthocyanin in Purple Potato Tubers ( *Solanum tuberosum* ).” Plant Direct 6: e418. 35865074 10.1002/pld3.418 PMC9289217

[mpp70069-bib-0053] Xu, L. , K. Yuan , M. Yuan , et al. 2020. “Regulation of Rice Tillering by RNA‐Directed DNA Methylation at Miniature Inverted‐Repeat Transposable Elements.” Molecular Plant 13: 851–863. 32087371 10.1016/j.molp.2020.02.009

[mpp70069-bib-0055] Yang, H. , Y. Zhao , N. Chen , et al. 2021. “A New Adenylyl Cyclase, Putative Disease‐Resistance RPP13‐Like Protein 3, Participates in Abscisic Acid‐Mediated Resistance to Heat Stress in Maize.” Journal of Experimental Botany 72: 283–301. 32936902 10.1093/jxb/eraa431

[mpp70069-bib-0056] Yang, X. , Y. Lu , F. Wang , et al. 2020. “Involvement of the Chloroplast Gene Ferredoxin 1 in Multiple Responses of *Nicotiana benthamiana* to *Potato virus X* Infection.” Journal of Experimental Botany 71: 2142–2156. 31872217 10.1093/jxb/erz565 PMC7094082

[mpp70069-bib-0057] Yin, L. , H. Chen , B. Cao , J. Lei , and G. Chen . 2017. “Molecular Characterization of *MYB28* Involved in Aliphatic Glucosinolate Biosynthesis in Chinese Kale (*Brassica oleracea var. alboglabra* Bailey).” Frontiers in Plant Science 8: 1083. 28680435 10.3389/fpls.2017.01083 PMC5478679

[mpp70069-bib-0058] Yin, Q. , S. Jiang , D. Li , et al. 2021. “First Report of *Epicoccum nigrum* Causing Brown Leaf Spot in Tea in Guizhou Province, China.” Plant Disease 106: 321.

[mpp70069-bib-0059] Yin, Q. , R. Yang , Y. Ren , et al. 2021. “Transcriptomic, Biochemical, and Morphological Study Reveals the Mechanism of Inhibition of *Pseudopestalotiopsis camelliae‐sinensis* by Phenazine‐1‐Carboxylic Acid.” Frontiers in Microbiology 12: 618476. 33859623 10.3389/fmicb.2021.618476 PMC8042141

[mpp70069-bib-0060] Yu, Y. , Z. Wu , and K. Lu . 2016. “Overexpression of the MYB Transcription Factor *MYB28* or *MYB99* Confers Hypersensitivity to Abscisic Acid in *Arabidopsis* .” Journal of Plant Biology 59: 152–161.

[mpp70069-bib-0061] Yu, Y. , Y. Zhang , X. Chen , and Y. Chen . 2019. “Plant Noncoding RNAs: Hidden Players in Development and Stress Responses.” Annual Review of Cell and Developmental Biology 35: 407–431. 10.1146/annurev-cellbio-100818-125218 PMC8034839 31403819

[mpp70069-bib-0062] Zhang, B. , H. Yang , D. Qu , Z. Zhu , Y. Yang , and Z. Zhao . 2022. “The MdBBX22‐miR858‐*MdMYB9/11/12* Module Regulates Proanthocyanidin Biosynthesis in Apple Peel.” Plant Biotechnology Journal 20: 1683–1700. 35527510 10.1111/pbi.13839 PMC9398380

[mpp70069-bib-0064] Zhang, Y. , Z. Zeng , H. Hu , et al. 2024. “MicroRNA482/2118 is Lineage‐Specifically Involved in Gibberellin Signalling via the Regulation of *GID1* Expression by Targeting Noncoding *PHAS* Genes and Subsequently Instigated phasiRNAs.” Journal of Plant Biotechnology 22: 819–832. 10.1111/pbi.14226 PMC10955497 37966709

[mpp70069-bib-0065] Zhao, S. , X. Wang , X. Yan , et al. 2018. “Revealing of the MicroRNA Involved Regulatory Gene Networks on Terpenoid Biosynthesis in *Camellia sinensis* in Different Growing Time Points.” Journal of Agricultural and Food Chemistry 66: 12604–12616. 30400742 10.1021/acs.jafc.8b05345

[mpp70069-bib-0066] Zhao, Y. , P. Chen , L. Lin , et al. 2011. “Tentative Identification, Quantitation, and Principal Component Analysis of Green Pu‐Erh, Green, and White Teas Using UPLC/DAD/MS.” Food Chemistry 126: 1269–1277. 25544798 10.1016/j.foodchem.2010.11.055 PMC4276396

