# Supplementary figures and images for: gma‐miR828a Negatively Regulates Resistance to Tea Leaf Spot Caused by Lasiodiplodia theobromae Through Targeting the CsMYB28– CsRPP13 Module
Source: Mol Plant Pathol. 2025 Mar 3;26(3):e70069. doi: 10.1111/mpp.70069 (PMC11876294; doi:10.1111/mpp.70069)

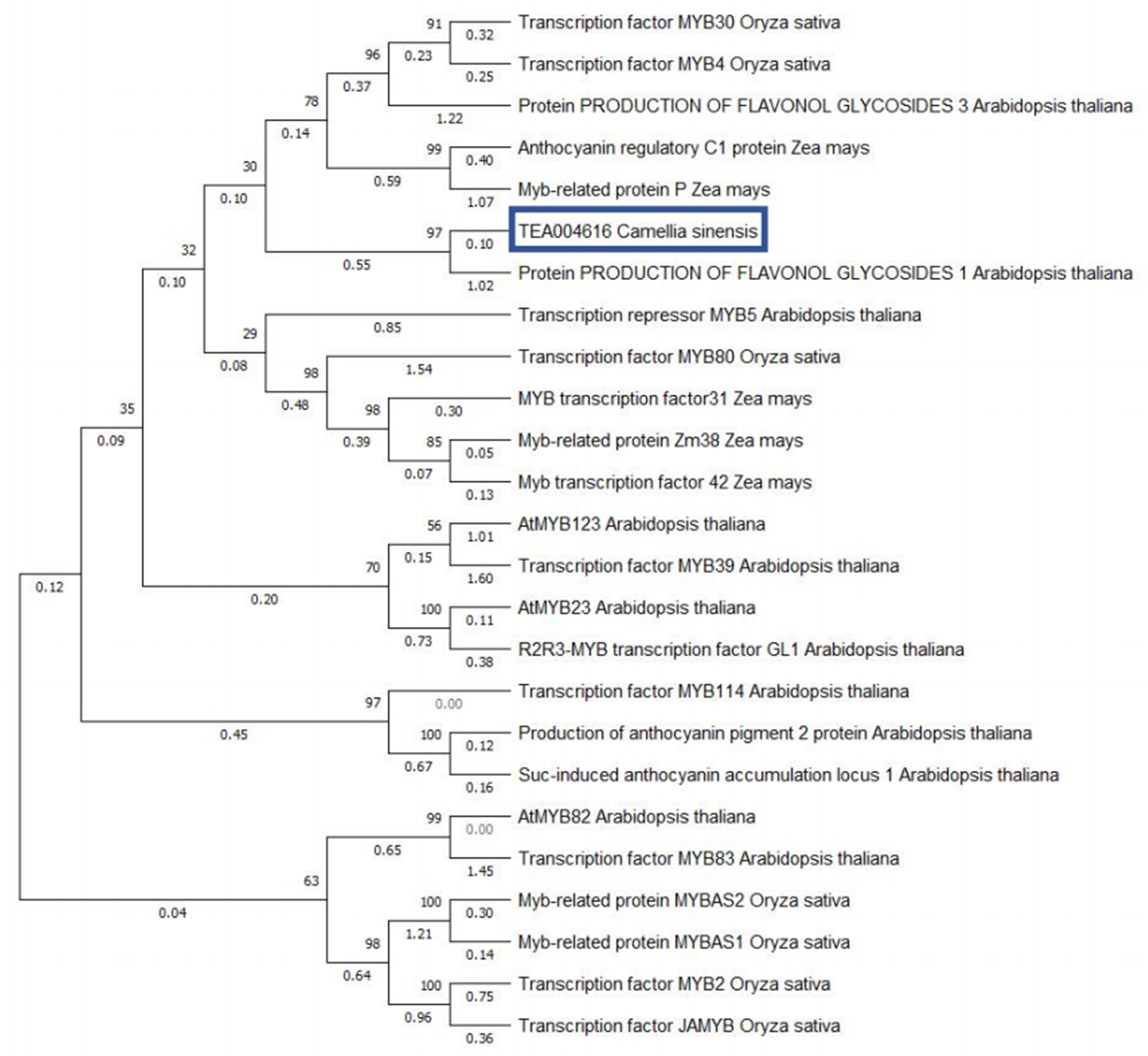

Supplement: Supplementary file 1 — Figure S1. A phylogenetic analysis of CsMYB28. [file MPP-26-e70069-s002.jpeg]

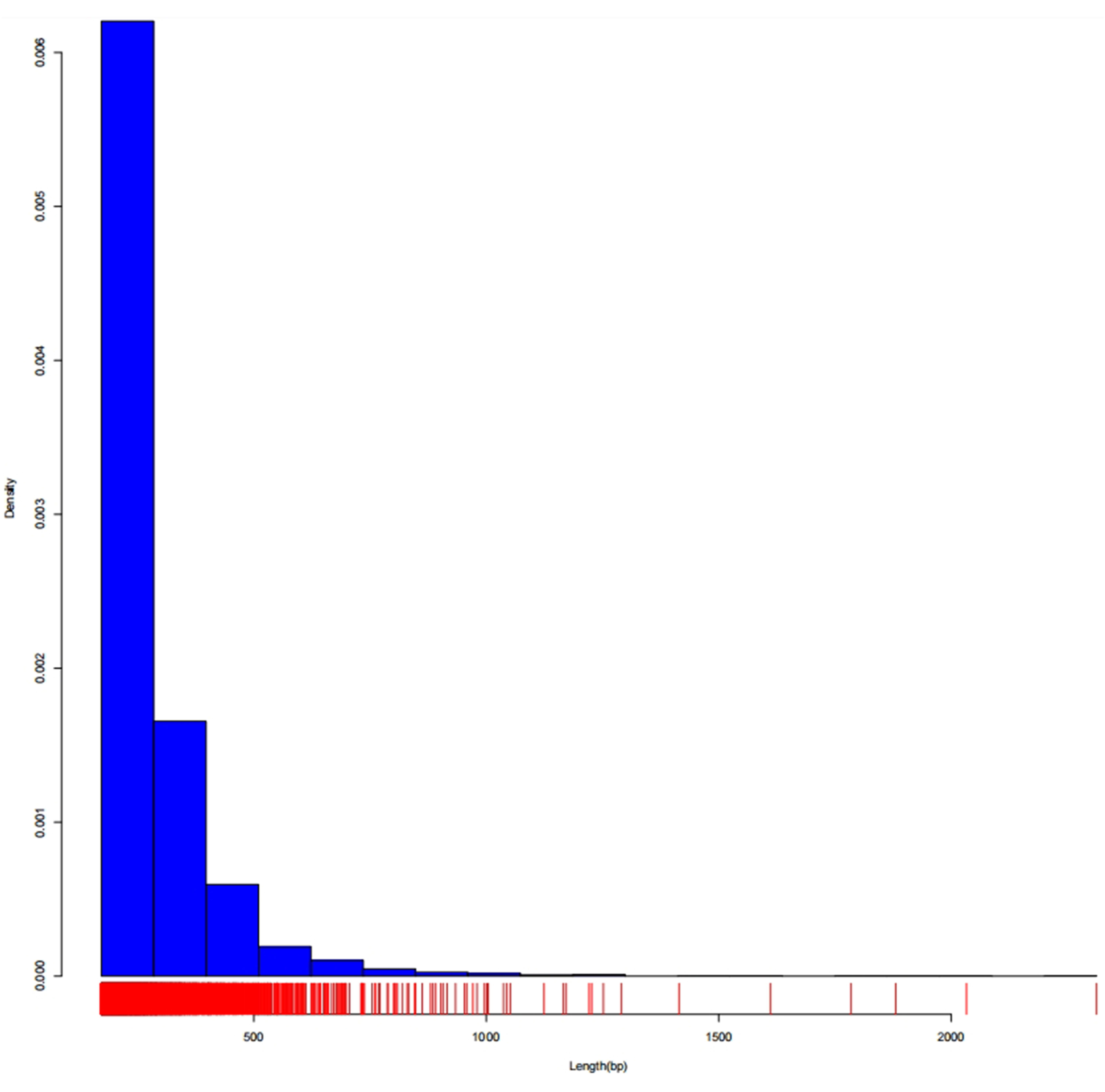

Supplement: Supplementary file 2 — Figure S2. Distribution of peak length by DAP‐seq. [file MPP-26-e70069-s003.jpeg]

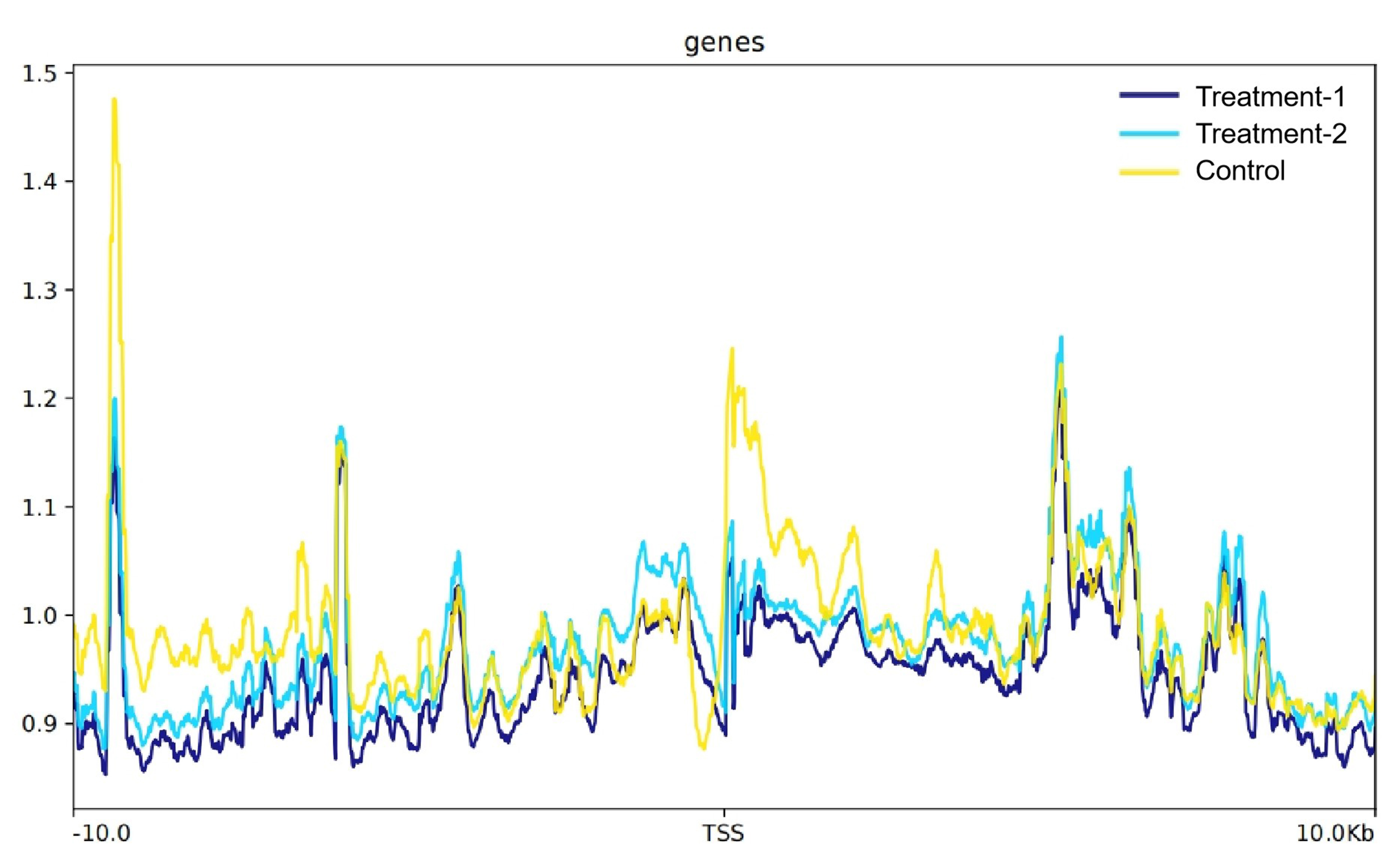

Supplement: Supplementary file 3 — Figure S3. The average abundance of reads near CsMYB28‐binding regions within the 10‐kb region upstream and downstream of the transcription start site (TSS) for CsMYB28‐binding regions. [file MPP-26-e70069-s005.jpeg]
